# Some Notes from the Olympia Motor Show

**Published:** 1919-11-15

**Authors:** 


### THE DOCTOR'S LIGHT CAR.

### Some Notes from the Olympia Motor Show.

MUCH has been lately written of the marvellous inovations and the glittering giants of the road to be seen at the present motor show, but it is probable that inspection of these recorded impressions is of small assistance to the busy practitioner urgently needing a professional car but unable to spare the time to come Londonwards and see for himself. The writer, perhaps more fortunate, was able to make a relatively complete tour of the thronging building and to leave no stone unturned in a search for the ideal doctor's car. The result of his final choice is immaterial to this note, but much of the information upon which the process of decision was founded may be of interest to those who share his desire for a medium-sized, coupé 12-h.p. vehicle which shall be reasonably priced, of smart, strong, lasting construction, and, above all, reliable. Obviously, makers of cars of the heavier types and heavier prices will have many customers among members of the medical profession, and of these vehicles there is no more to be said than that the already established British makers have more than lived up to their reputation by producing some superb models. In the ranks of the lighter cars, there have, however, appeared a number of newcomers with excellent companies and workshops behind them which should guarantee their material, delivery, and spare-part service, in a manner making them more than serious rivals to the new products of the pre-war firms. It is to this section of the show that we will confine our attention, in that into this class will probably fall the motoring requirements of the great majority of medical men.

### ELECTRIC LIGHTING AND STARTING.

This combined electrical equipment appears as standard on practically every make, and, without exception, the construction is so good and the parts so neatly, yet strongly, embodied in the chassis that, apart from the successful demonstrations seen, one can with confidence dispose of the old-time indictment of unreliability. The controls beautifully fitted flush in the dashboard and of perfect accessibility from the driving seat will be a boon to the owner-driver. The small sets used on the 12-h.p. car do not need long runs to keep their batteries charged and the customary distances and times covered by the practitioner should provide ample power for both lighting and engine turning. In any case a pair of oil sidelights can be carried in the luggage space or dickey, and a duplicate tail lamp is a simple matter. The doctor should certainly employ an electrically equipped machine.

### COACH WORK AND BODIES.

It is useless to deny that the brasswork and stout panelling of the pre-war car are now conspicuous by their absence, and that nickel fittings will inevitably suffer from weather exposure, but otherwise the standard of general turn-out is very good and, in those machines mentioned in detail later, it is excellent. Ease of cleaning and general appearance are greatly improved by the removal from *outside* the cars of all superfluous fittings and difficult angles, the closing in by metal of all spaces between body, footboard, and wheels, reducing flying mud to a minimum, and the frequent use of simple pressed steel or disc wheels. Aluminium enters tremendously into the new constructions, playing a great part in keeping up a smart appearance and the many panels and fittings made of solid, unrustable white metal should prove invaluable to the same end. Some of the cars have not a tenth of the paintwork which our previous models displayed. Simplicity of external line and fittings is a keynote and a tendency which spells for much saved time.

### ENGINE AND CHASSIS.

Apart from one or two radial cylindered and twin-cylindered models, and some with curiously complicated chassis springing, one notes that the main principles of engine and drive construction in the 12-h.p. models have undergone no great change. Compactness, strengthening of individual parts, elimination or reduction of points of weakness, and, above all, accessibility, have been, however, amazingly improved. One has heard criticisms of the apparently unnecessary size and height of the modern radiators and bonnets, but if he who has spent many an hour hopelessly groping with an engine which almost completely fills its allotted car space, will lift the panel of one of the new productions he will realise what advantages this extra room can give. Lubrication methods are certainly improved. Far less is now left to chance, oil is guided deliberately from the main reservoir to every part necessary and as little reliance as possible placed on the frequency of activities with a small oil-can. A point that many of us have experienced is the strain and corresponding wear set up by a brake directly gripping some part of the driving shaft. Very great and irregular tension strains are set up in this way, and it is most satisfactory to note that in almost every case *both* hand and foot-brakes now act on big drums on the back wheels. Also how many experienced premature back-axle or crown-wheel trouble in the lighter makes of car? At Olympia we see at least a 50 per cent. increase in the size and stoutness of these parts in the modern vehicle, an inestimable advantage when driving over the abominable road surfaces with which the country seems liable to be cursed since the advent of heavy road transport.

### SOME TYPICAL EXAMPLES.

In making mention of those cars which are particularly worthy of the doctor's inspection one may omit the already well-known makes, prominently displayed as they are. On the whole simple modernisation has been carried out along such lines as already mentioned, but near by and far more appealing at the moment, are their potential rivals. In these, recent experience, thought, and adaptation of the immense factories and shops which sprang up during the war have combined to give the country some truly excellent products.

Particularly one noticed the 11.5-h.p. Beardmore chassis (Beardmore Motors, Ltd., Great Portland Street, W.), which is of pleasingly solid construction and finish and sells at £365, or as a completely equipped two-seater at £425, the *coupé* model being a few pounds more. Behind this manufacture is the old-established engineering firm of William Beardmore and Co., Ltd., and from inspections and demonstrations one would class this car as one of the finest of its type in the show. Certainly it embodies all the soundest principles of car manufacture, and in outline may be described as 4-cylinder, detachable steel wheels, wheel base 8 ft. 6 in., completely electrically equipped, with an excellent set of dashboard indicators, speedometer, etc. It is an extremely smart and graceful car, eminently suited to a doctor.

Another noteworthy model is the 11.9-h.p. Albert 4-cylinder car, manufactured by Adam, Grimwaldi and Co., Ltd., Vauxhall, London. It is somewhat larger than the first mentioned, having a wheel base of 9 ft.

6 in. and a chassis price of £469 7s. 6d. The *coupé* model on exhibition is a very smart production and sells at £665 10s., the two-seater at £534 10s., equipment being absolutely complete in every way. The C.A.V. electric units and the miniature Rolls type radiator give a most pleasing appearance, and mechanically one has little fault to find. A car to which attention should certainly be given is the 11.9-h.p. Bean, of A. Harper, Sons & Bean, Ltd., Dudley, Worcs., with a chassis weight of 14 cwt., length 8 ft. 6 in., and price £325. The complete car prices are for the open two-seater £425 and for the *coupé* £500. It is undoubtedly an excellent proposition, and is apparently backed by a first-rate spare-part service. Elsewhere notice the Rover 12-h.p., a beautiful *coupé* at £800, the Secqueville Hoyau at £550, the Charron-Laycock 10-h.p. *coupé* at 540 guineas.

On the whole one has attempted to judge with regard to value, rather than mere excellence alone. If price be but a small consideration, then Olympia doubtless holds the ideal for most of us, but for the range up to a £600 *coupé* price the doctor will find those cars mentioned more than hard to beat.

